# hiPSC-derived neural stem cells from patients with schizophrenia induce an impaired angiogenesis

**DOI:** 10.1038/s41398-018-0095-9

**Published:** 2018-02-22

**Authors:** Bárbara S. Casas, Gabriela Vitória, Marcelo N. do Costa, Rodrigo Madeiro da Costa, Pablo Trindade, Renata Maciel, Nelson Navarrete, Stevens K. Rehen, Verónica Palma

**Affiliations:** 1 Laboratory of Stem Cells and Development, Universidad de Chile, Santiago, Chile; 2 D’Or Institute for Research and Education (IDOR), Rio de Janeiro, Brazil; 3 Institute of Biomedical Sciences, Federal University of Rio de Janeiro, Rio de Janeiro, Brazil; 4 Universidad de Chile Clinical Hospital, Región Metropolitana, Chile

## Abstract

Schizophrenia is a neurodevelopmental disease characterized by cerebral connectivity impairment and loss of gray matter. It was described in adult schizophrenia patients (SZP) that concentration of VEGFA, a master angiogenic factor, is decreased. Recent evidence suggests cerebral hypoperfusion related to a dysfunctional Blood Brain Barrier (BBB) in SZP. Since neurogenesis and blood-vessel formation occur in a coincident and coordinated fashion, a defect in neurovascular development could result in increased vascular permeability and, therefore, in poor functionality of the SZP’s neurons. Here, we characterized the conditioned media (CM) of human induced Pluripotent Stem Cells (hiPSC)-derived Neural Stem Cells of SZP (SZP NSC) versus healthy subjects (Ctrl NSC), and its impact on angiogenesis. Our results reveal that SZP NSC have an imbalance in the secretion and expression of several angiogenic factors, among them non-canonical neuro-angiogenic guidance factors. SZP NSC migrated less and their CM was less effective in inducing migration and angiogenesis both *in vitro* and *in vivo*. Since SZP originates during embryonic brain development, our findings suggest a defective crosstalk between NSC and endothelial cells (EC) during the formation of the neuro-angiogenic niche.

## Introduction

Schizophrenia is a debilitating mental disorder that affects 1% of the world population and is characterized by positive and negative behavioral, cognitive, and psychological symptoms^1^. Physiologically, schizophrenia patients (SZP) have brain connectivity deficiencies, neurotransmitter dysfunctions, loss of gray brain matter, and an abnormal distribution of neurons in the prefrontal cortex. To date, schizophrenia has no cure and pharmacological treatments are only partially efficacious. Schizophrenia has been described as a multiple-etiology disease, originated during nervous system’s embryonic development, despite the fact that it is diagnosed during adolescence^2,3^. At present, the mechanisms that trigger and predict the evolution of this disease remain largely unknown. Recent literature proposes the importance of mutations in genes associated with neuronal migration and synaptic plasticity^4^.

The neural and vascular networks have been described as having a mutual relationship based on their morphological similarity, co-dependence, and the ability of molecules to regulate both their formations during development^5^. The latter has led to the birth of the concept of neuro-angiogenesis, which details the coordinated development of neurons (neurogenesis) and formation of new blood vessels (vasculogenesis and angiogenesis). Almost as soon as the central nervous system forms, the neural tube (NT) begins communicating with the surrounding mesodermal tissue—where angioblasts and endothelial cells (EC) reside. Vasculogenesis in the developing brain starts when Neural Stem Cells (NSC), and later Neural Progenitor Cells (NPC), from the NT recruit angioblasts and EC to form the perineural vascular plexus (PNVP)^6–8^. The main derived NT signal corresponds to Vascular Endothelial growth factor A (VEGFA), the master regulator of blood vessel formation^9,10^. Although these vessels proliferate and migrate within the NT, the NT itself is developing via neurogenesis^6,7,11,12^. NSC differentiation and migration during development depend on the trophic support provided by early blood vessels^13^. Both the vascular and nervous systems are regulated by a combination of attractant and repulsive molecular signals. Changes in the factors present in the neuro-angiogenic niche can have profound consequences in proper brain development and the vascular network associated with it^14,15^. To date however, the link between neuro-angiogenesis alterations in brain formation and neurodevelopmental diseases are not well studied.

Interestingly, researchers have provided strong evidence linking SZP to vascular abnormalities and blood brain barrier dysfunction^16^. In particular, VEGF blood levels in adult SZP have been shown to be lower than in control subjects^17^. Furthermore, *VEGF* and *VEGFR2* (*KDR*) mRNA were demonstrated to be decreased in the post-mortem dorsolateral prefrontal cortex of SZP^18,19^. Levels of fibroblast growth factor-2 (FGF-2), another important angiogenic factor, are altered in peripheral blood of SZP patients^20^. Brain biopsies of SZP have been found to have higher levels of Semaphorin 3A (SEMA3A), a chemorepellent involved in axon guidance and described as being anti-angiogenic^21–23^. Note that all of these data were obtained using adult or post-mortem tissues. There are scarce biological studies examining the mechanisms underlying neurovascular abnormalities in SZP, and they are far from being consensual^24^. To date, the status of vasculogenic and angiogenic signals and their relevance during embryonic development of SZP patients remain virtually unknown.

NSC derived from human induced pluripotent stem cells (hiPSCs) conserve the genetic diversity of donors and can recapitulate neurogenesis in vitro, thus serving as a model for the study of various neurological diseases^25–27^. Indeed, researchers have observed that NSC and NPC from SZP hiPSC preserve the phenotypic characteristics of schizophrenia, such as increased oxidative stress, mitochondrial dysfunction, synaptic abnormalities, differences in migration, and response to drugs that are widely used for treatment^28–32^. Since the disease originates during brain development, here we evaluated possible alterations in the process of neuro-angiogenesis using hiPSC-derived NSC obtained from three subjects diagnosed with schizophrenia spectrum (SZP NSC). In particular, we evaluated whether SZP NSC could induce an impaired angiogenesis due to an imbalance in the secretion of angiogenic factors.

Our results suggest that impaired neural function in SZP can in part be explained by a deficiency in angiogenesis induction by SZP NSC. We demonstrate the importance of the vasculature of the developing neural stem cell niche and that its dysfunction likely contributes to impaired neurogenesis in the SZP brain.

## Materials and methods

### SZP fibroblast collection and culturing

Skin fibroblasts from individuals were obtained under sterile conditions. Briefly, 10-mm full thickness skin cut biopsies were collected from patients’ necks. Biopsies were chopped into 2 × 2 mm^2^ pieces, plated in T75 flasks, and maintained in DMEM/F12 medium supplemented with 10% fetal bovine serum (FBS) and 20% ciprofloxacin. After 14 days, fibroblasts were detached from flasks using TrypLE^TM^ Express (Invitrogen, Carlsbad, CA, USA) and maintained in DMEM/F12 medium supplemented with 10% fetal bovine serum (FBS) without antibiotics.

### Generation of hiPS cells

SZP cell lines used in this study were obtained from three subjects diagnosed with the schizophrenia spectrum (Supplemental Table 1). To decrease genetic heterogeneity among cell lines^33^ two of these patients are siblings. Patient 1 (GM23760B) is a male who presented paranoid schizophrenia symptoms, while his sister (GM23761B) was diagnosed with schizoaffective disorder and has a history of drug abuse (Brennandet al.^31^, available at Coriell). The third subject is a male patient, non-relative to the previous ones, who also presented paranoid schizophrenia symptoms (EZQ4)^34^. Three control cell lines were used: one cell line was obtained from a female subject (GM23279A, available at Coriell); while the other two hiPS cell lines were reprogrammed at the D’Or Institute for Research and Education and were derived from male subjects (CF1 & CF2)^34^. For reprogramming, fibroblasts were plated into 6-well plates. After 1–2 days in culture, viral infection of fibroblasts was carried out according to the manufacturer’s protocol. After 24 h of viral incubation, the medium was replaced and the cells were maintained in standard culture conditions for 6 days, until the first hiPSC colonies appeared. Next, pluripotent-like cell colonies were transferred to Matrigel-coated plates and cultured using mTeSR media (Life Technologies, Carlsbad, CA, USA) for ~28 days; plates were observed and checked every day under a microscope. After 2–3 weeks of viral transduction, colonies reached a size that allowed further passage and expansion.

### In vitro neural differentiation

All six human hiPSC lines were adapted to E8 medium (Thermo Fisher Scientific, Carlsbad, CA, USA) for at least four consecutive passages, and cells were later split. After 24 h of splitting the cells, we maintained them in Pluripotent Stem Cells (PSC) Neural Induction Medium (Thermo Fisher Scientific, Carlsbad, CA, USA), which contained Neurobasal medium and PSC supplement, according to the manufacturer’s protocol. Medium was changed every other day for 7 days, during which initial NSCs split and expand with Neural Induction Medium (NEM, Advanced DMEM/F12 and Neurobasal medium (1:1) with Neural Induction Supplement; Thermo Fisher Scientific, Carlsbad, CA, USA).

### Neurosphere (Nsp) formation and migration assay

NSC cells were grown in NEM supplemented with 1×N2 and 1×B27 supplements under rotation at 90 rpm, and medium was replaced every 4 days. Nsp were seeded in poli-l-ornitin/laminin coated (10 µg/ml and 2.5 µg/ml respectively) 96 well plates. Images were acquired with the Operetta high-content imaging system, every 30 min for 48 h. Neurosphere area and axonal migration area were measured using the software Image J (NIH, USA).

Establishment of hiPSC, and derivation of NSC and Nsp lines were carried out in accordance to international standards and with the approval of the research ethics council (CAAE: 32385314.9.0000.5249).

### Morphometry and cell division analysis

NSCs were plated in Geltrex coated 96-well μClear plates (Greiner, Frickenhausen, Germany) in triplicate and monitored for 20 h using the live-cell monitoring chamber coupled to a high-content screening microscope (Operetta, Perkin Elmer, Waltham, CA, USA). Phase-contrast images were acquired every 15 min for 12 h and cells were automatically counted and morphologically analyzed using Harmony 5.1 (Perkin Elmer, Waltham, CA, USA). Each individual cell in each phase-contrast image was tracked for 12 h. Every time a cell divided, the software counted a new cell generation. The total number of generations is normalized to the total number of cells. This value therefore represents the cell culture doubling time.

### Immunofluorescence

After live-cell image acquisition, cells were fixed with a 4% paraformaldehyde solution, permeated with a 0.3% Triton-X (Sigma, St. Louis, MO, USA) solution, blocked with a 3% BSA solution, and incubated with anti-Nestin and anti-Pax6 antibodies (Supplementary Table 2). Subsequently, cells were incubated with secondary antibodies (Supplementary Table 2) for one hour. Nuclei were stained with 0.5 μg/mL 4′-6-diamino-2-phenylindole (DAPI) for 5 min. Images were acquired with the Operetta high-content imaging system with a ×20 objective, high numerical apertures (NA) (PerkinElmer, Waltham, CA, USA). The total number of cells was calculated using the number of DAPI stained nuclei. Data analysis was performed using the high-content image analysis software Harmony 5.1 (PerkinElmer, Waltham, CA, USA). Twenty different fields from triplicate wells per experimental condition were used for quantification and morphometry analysis.

### Conditioned medium (CM) collection

NSC cultures were grown in 60 mm plates to 80% confluency and treated with fresh NEM (Advanced DMEM/F12 and Neurobasal medium (1:1) with Neural Induction Supplement; Thermo Fisher Scientific, Carlsbad, CA, USA). Nsp were cultured in agitation for 4 days as described before and medium was changed with fresh NEM. Conditioned medium was collected 48 h after medium replacement from cultures and then fast frozen in liquid nitrogen, and stored at −80 °C until further use. At the day of collection, there were ~1 million cells per ml of CM.

### Angiogenic proteome profiling

The presence of angiogenic factors was evaluated in NSC and Nsp CM with a Proteome Profiler Human Angiogenesis Array kit (Catalog # ARY007, R&D Systems Inc., Minneapolis, MN, USA) according to the manufacturer’s instructions. In total 1 mL of CM from each condition (3 Ctrl NSC, 3 SZP NSC, 3 Ctrl Nsp and 3 SZP Nsp) was assayed. Spots were detected by enhanced chemiluminescence and intensity was quantified by densitometry using the software ImageJ (NIH, USA). The pixel intensity of each factor (in duplicate) was normalized to that of three internal controls provided by the assay. Each assay was performed in duplicate.

### Gene expression analysis via qPCR

Total RNA was obtained from NSC (3 Ctrl NSC and 3 SZP NSC) by phenol-chloroform extraction using RNAsolv (Omega Bio-Tek, Norcross, GA, USA). cDNA was then synthesized using 1 μg of RNA and a M-MLV reverse transcription kit (Promega, Madison, WI, USA), according to the manufacturer’s instructions. Relative expression was assessed by qPCR (Agilent Technologies Thermocycler, Santa Clara, CA, USA) using specifically designed primers as indicated in Supplementary Table 3. Data was analyzed by calculating the expression fold change via 2^-ΔΔCt^, and gene expression was normalized to that of three reference genes (*GAPDH*, *B2M*, and *18S*).

### Western blot

Protein extracts were obtained from NSC CM (3 Ctrl NSC CM and 3 SZP NSC CM) via methanol-chloroform extraction. Briefly, 500 µl of cold methanol and 125 µl of cold chloroform were added to 1 ml of CM, vortexed and spun down at 1400 × *g* for 5 min at 4 °C. The white interphase was resuspended in 25 µl of Extraction buffer composed of 2% SDS, 10% Glycerol, 50 mM Tris–HCl pH 6.8, and protease inhibitor (Catalog # 88265; Thermo Scientific, Waltham, MA, USA). Protein extracts were stored at −20 °C. We pipetted 60 µg of protein into each gel lane, separated in 8–12% SDS–PAGE, and transferred to nitrocellulose membranes. Membranes were incubated overnight with primary antibodies for SEMA3 and SLIT2 (Supplementary Table 2). Membranes were washed with Tris buffer saline (TBS) with 0.1% Tween, and incubated (1 h, 22 °C) in 0.1% TBS-Tween containing horseradish peroxidase-conjugated goat anti-mouse secondary antibody. Protein bands were visualized using enhanced chemiluminescence (ECL; Amersham Biosciences, Little Chalfont, UK) and quantified by densitometry using Image J (NIH, USA).

### Endothelial cell tube formation assay

To assess the angiogenic potential of CM from different NSC and Nsp batches (3 Ctrl NSC, 3 SZP NSC; 3 Ctrl Nsp and 3 SZP Nsp), we carried out tubule formation assays using human umbilical cord endothelial cells (HUVEC), as previously described^35^. Briefly, umbilical cord veins were washed with a warm phosphate buffered saline solution (PBS: 136 mM NaCl, 2.7 mM KCl, 7.8 mM Na_2_HPO_4_, 1.5 mM KH2PO4, pH 7.4). Endothelial cells were isolated via digestion with 0.2 mg/mL collagenase and recovered with medium 199 (M199). Cells were seeded onto 1% gelatin coated dishes and cultured in primary cell medium (PCM, M199 plus 10% NBCS, 10% FBS, 3.2 mM l-glutamine and 100 U/mL penicillin-streptomycin) at 37 °C, 5% CO_2_. The medium was changed every two days until 80% confluence was reached. All HUVEC primary cultures were used between passages two to five.

Cells (55.000/well) were seeded onto solid growth factor-reduced Matrigel (BD Biosciences, San Jose, CA, USA) in 96-well plates with the following stimuli: harvested 48 h NSC CM, Nsp CM, NEM, Endothelial Growth Medium (EGM-2; Lonza, Verviers, Belgium; used as positive control), or Endothelial Basal Medium (EBM, Clonetics, Walkersville, MD, USA; negative control). A humanized monoclonal antibody that binds to VEGFA (100 µg/ml Bevacizumab, Roche Diagnostics GmbH, Mannheim, Germany) was used to evaluate the contribution of VEGFA to NSC CM-induced angiogenesis; 50 ng/ml of recombinant VEGFA was used as control. Each stimuli was assessed in triplicate. After four hours of incubation, images from five different fields were taken per well. Tubular networks were quantified by counting the number of branching points and new tubules formed using ImageJ (NIH, USA).

### Wound healing assay

HUVEC or NSC were seeded onto a 1% gelatin coated 12-well culture plate until 100 % confluence was reached. To evaluate the migration of cells, we conducted a scratch assay. Briefly, the cell monolayer was scratched using a 200 μl sterile tip. Conditioned media, collected from 48 h NSC cultures, were used on HUVEC. Photographs of the wound were taken at the initiation of incubation (time 0) and after eight hours of incubation. The scratched zone area was measured using Image J; data were presented as the percentage of wound closure compared to initial wound area.

### Chicken chorioallantoic membrane (CAM) assay

For an in vivo evaluation of the angiogenic inductive potential of NSC (3 Ctrl NSC and 3 SZP NSC), a CAM assay was performed as previously reported, with minor modifications^36^. Briefly, fertilized chicken eggs (Rock iso, Agricola Chorombo, Chile) were incubated at 38.5 °C with constant 75% humidity. At embryonic day 1 (E1), 2 mL of albumin was extracted from each egg; a round window (2 cm^2^) was created on E4. A home-made Bio cellulose scaffold (sham) of bacterial origin (6 mm diameter) was filled with 100 µl of medium to be assed: NSC CM, NEM, 100 µg VEGFA (as positive control), and PBS (as negative control). On E8, the CAM vasculature was photographed; subsequently, each experimental condition scaffold was placed on top of the CAM; for each condition 10 eggs were used. Control eggs (Sham) did not have scaffolds in order to assess its impact on angiogenesis. On day E12, white cream was injected under the CAM before photographing every egg, in order to improve the visualization of the vessels. Photographs were taken with a digital camera HD IC80 (Leica, Heidelberg, Germany) and the number of vessels within a 6-mm radius of the scaffold were counted. A humanized monoclonal antibody that binds to VEGFA (100 µg/ml Bevacizumab, Roche Diagnostics GmbH, Mannheim, Germany) was used to evaluate the contribution of VEGFA to NSC CM-induced angiogenesis. The number of vessels that enter the scaffold was counted to determine the angiogenic score, using ImageJ software (NIH, USA).

### Statistical Analysis

All statistical analyses were performed using Graphpad Prism 7.03 (GraphPad Software Inc). Normality was assessed with D’Agostino-Pearson test. We used Mann–Whitney U and Kruskal–Wallis tests of comparison. All values are expressed as mean ± S.D. Statistical significance was set at *p* < 0.05.

## Results

### Profiling hiPSC-derived NSC from SZP and Ctrl subjects

**Fig. 1:**
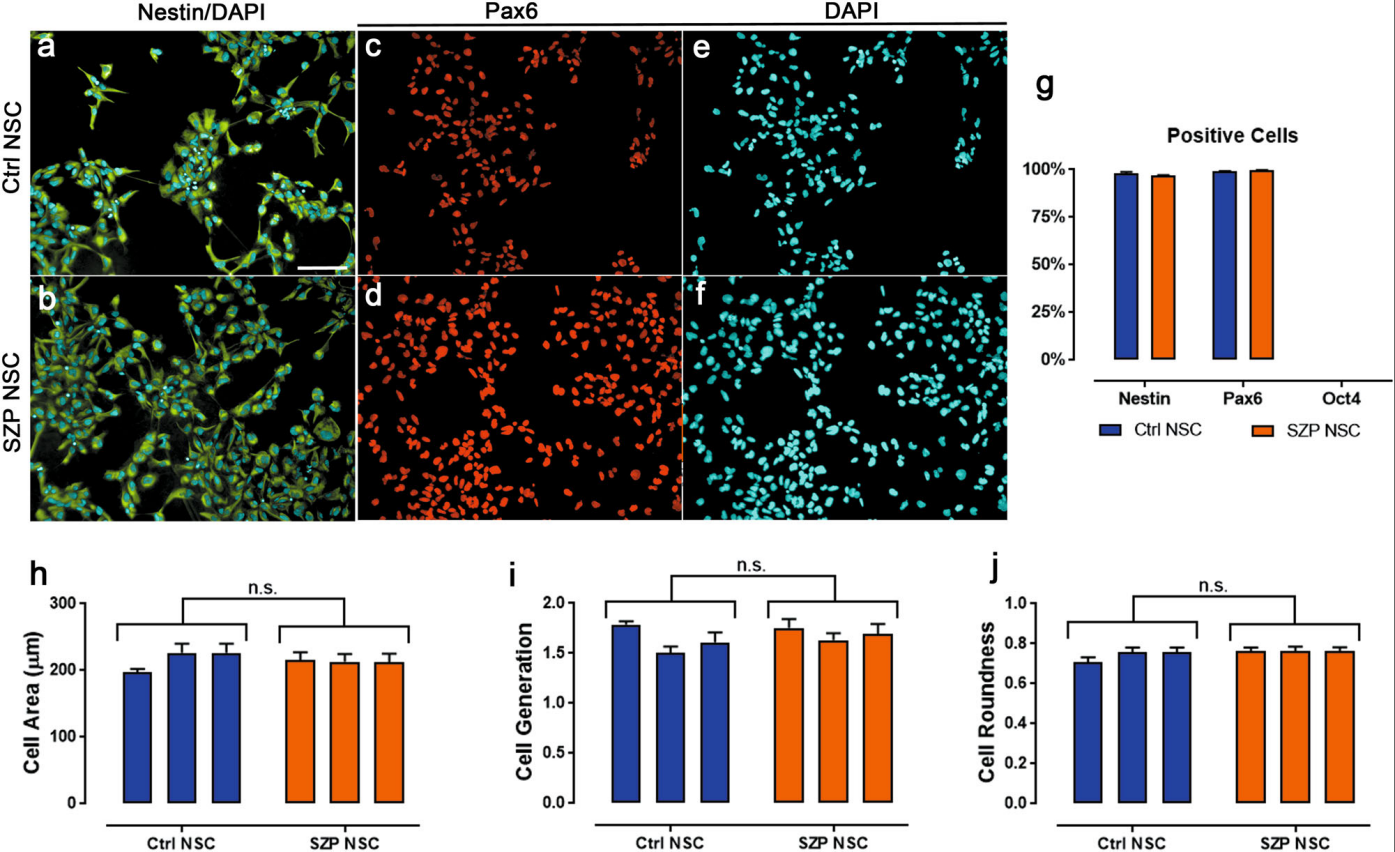
Profiling of NSC derived hiPSC obtained from Ctrl and SZP. Immunostaining for Ctrl (**a**, **c**, and **e**) and SZP (**b**, **d**, and **f**) NSC specific markers; (**a** and **b**) Nestin (green) and nuclear marker DAPI (blue), (**c** and **d**) Pax6 (red) and corresponding nuclear marker DAPI (**e** and **f**); Scale bar = 100 um. Representative images from *N* = 3 (3 Ctrl NSC and 3 SZP NSC). **g** Percentage of positive cells, with 98% (±0.003) and 97% (±0.01) Nestin positive cells for Ctrl and SZP NSCs respectively; 99% (±0.002) and 99.7% (±0.002) Pax6 positive cells for Ctrl and SZP NSCs respectively. No Oct4 positive cells were detected in NSC of Ctrl and SZP. Data is shown as mean ± SD from *N* = 3. **h** Cell division was monitored for 12 h and no significant differences in proliferation were found between Ctrl and SZP NSC. Data for each cell line (Ctrl #1, #2, #3 and SZP #1, #2, #3) is shown in a correlative order control and graphed as mean ± SD. **i**, **j** Morphological parameters such as cell area (**i**) and cell roundness (**j**) did not show detectable differences among Ctrl and SZP groups. Data for each cell line (Ctrl #1, #2, #3 and SZP #1, #2, #3) is shown in a correlative order control and graphed as mean ± SD

All NSC derived from control and SZP exhibited clear Nestin and Pax6 staining, the two most well characterized markers for NSC. There was no phenotypic difference in Nestin and Pax6 expression between Ctrl and SZP NSC (Fig. 1a–f). Nearly 99% of all counted cells were positive for both markers; we observed no difference in the number of positive cells when comparing control and SZP NSC (Fig. 1g). Morphologically, control and SZP NSCs were similar in terms of cell area (~200 μm^2^ for all cell lines), roundness (approx. 0.7 for all cell lines) (Fig. 1i, j), and generation of new cells (1.6×the number of seeded cells) (Fig. 1h). All cell lines tested were negative for Oct4, a pluripotency marker (Fig. 1g).

Prior research has shown hiPSC-derived SZP NPC to have defective migration^30^. In agreement with these results, we performed a neurosphere (Nsp) migration assay with our SZP NSC-derived Nsp (Supplementary Figure 1a) and found that although the SZP Nsp were similar in growth area, compared to Ctrl Nsp (Supplementary Figure 1b), there was a decrease in their axonal migrating area (Supplementary Figure 1c, Supplementary Video 1, Supplementary Video 2).

These results demonstrate that all cell lines tested possess the main NSC characteristics, that Ctrl and SZP NSC are morphologically and generatively similar, and that SZP NSC used in this work describe characteristic migration deficiencies.

### NSCs secrete angiogenic proteins

Numerous studies have proposed a mutualistic relationship between vascular and neural networks^14,37^. These propositions, however, are based on demonstrating the instructive role of the vasculature secreting soluble factors that promote self-renewal and proliferation of NSCs. More recent studies point to the ability of molecules to both regulate the formation of vasculature and influence neurogenesis during development^5,38^.

Since NSCs participate in the recruitment of EC and brain vessel maturation during development^39^, we first decided to analyze the presence of canonical angiogenic factors in the hiPSC derived NSCs CM/secretome and, secondly, to explore CM potential impact on angiogenesis. Three NSC were grown for 48 h in serum free media and the CM was collected for analysis through a proteome array. Of the 55 canonical angiogenic factors we assessed, we detected 20 in the NSC secretome. Of these, 14 are pro-angiogenic and six are anti-angiogenic proteins. Table 1 describes the main roles of each factor, and reveals that proteins secreted by Ctrl NSC participate in several angiogenic processes. We specifically identified factors related to the early stages of vasculogenesis (VEGFA), vessel stabilization (Ang-1, Tsp-1, MCP1), vessel destabilization and sprouting (Ang-2, uPA, PAI-1), as well as several proteins that commonly participate in EC migration and proliferation. Since all these factors are secreted into the neuro-angiogenic niche during brain morphogenesis, we also investigated whether these proteins also have roles within the context of neurogenesis (Table 1). Interestingly, we found that most of the secreted molecules do have a direct impact in neurogenesis, NSC proliferation, and/or migration. Some of these molecules (e.g., DPP IV, Prolactin, PTX3, TIMP1) are also involved in neuroprotection and post-insult neurogenesis; however, their specific effect, if any, during development remains unknown.

**Table 1:** Dual role of angiogenic molecules secreted by Ctrl hiPSC derived NSC

Molecule	Vascular system	Nervous system
Angiogenin	Wound healing; EC migration, invasion, proliferation, and formation of tubular structures^46^	Neuroprotection trough astroglia^47^
Ang-1	Vessel stabilization; EC differentiation; neovascularization^59,60^	Neuroprotection; neurogenesis.^61^
Ang-2	Vascular destabilization; angiogenesis^59^	Cortical neurogenesis; radial glia migration^62^
DPP IV	Vascular remodeling; Y2/Y5-mediated angiogenesis^63^	Post-stroke repairing^64^
Endothelin-1	EC and VSM proliferation^65^	Neuronal communication^66^
IGFBP-2	EC production and secretion of VEGFA^53^	Neurogenesis^67^
IGFBP-3	EC survival^55^	Inhibition of NPC proliferation^68^
IL-8	EC survival; differentiation^69^	NSC death; chemotaxis^70^
MCP-1	Chemotaxis; VSM recruitment^71^	NSC migration^72^
PDGF-AA	Promotion of VEGFA expression^51^	NPC differentiation into oligo-lineage^73^
PlGF	Regulation of pathological angiogenesis^74^	Neuroprotection; V-SVZ proliferation^75,76^
Prolactin	Pro- or anti-angiogenesis activity depending on isoforms^77^	Neurogenesis; neuroprotection^78^
uPA	Promotion of vascular permeability; EC proliferation and migration^52^	Neuronal migration; neuritogenesis; neuroprotection after injury^79^
VEGFA	EC survival, proliferation and migration; induction of blood vessel growth^80^	Neurogenesis; NSC recruitment^81,82^
Endostatin	Inhibition of EC proliferation^56^	Inhibition of neurite outgrowth and neuronal migration^83^
PTX3	Antagonism of FGF2 signaling^84^	Neurogenesis after cerebral ischemia^85^
PAI-1	Inhibition of uPA. Inhibition EC migration^86^	Neuron survival^87^
PEDF	Inhibition of physiological and pathological angiogenesis^88^	NSC renewal^89^
TIMP-1	Inhibition of EC migration^90^	Neurogenesis after ischemia^91^
TSP-1	Inhibition of EC migration, proliferation and survival^92^	Maintenance of the adult NPC pool; neuronal differentiation^93^

### Deregulation of neuro-angiogenic proteins expression in SZP NSC

**Fig. 2:**
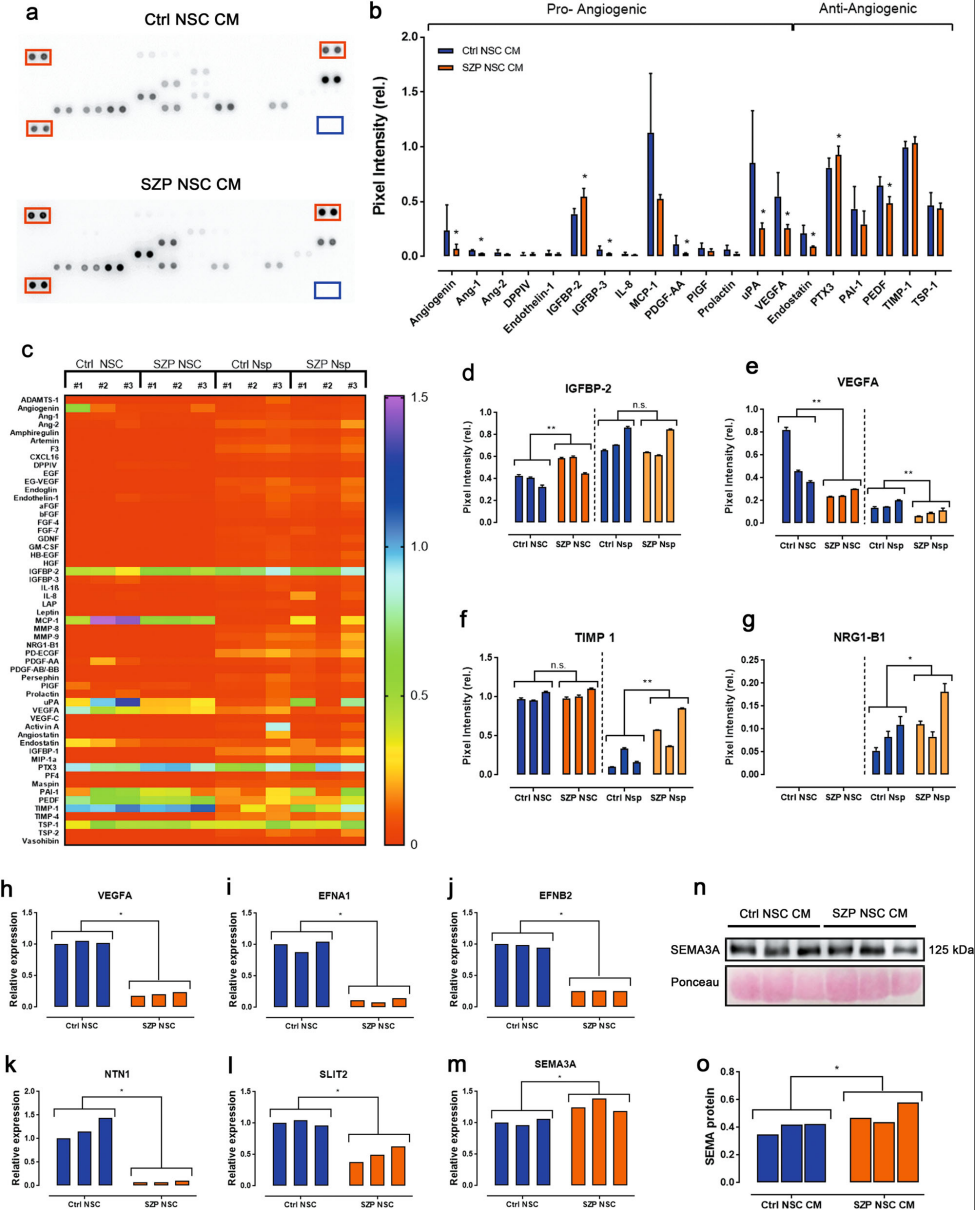
SZP NSC present an imbalance in neuro-angiogenic factor secretion. **a** Representative pictures of angiogenic profile secretome from Ctrl NSC and SZP NSC. Red boxes indicate positive internal control spots; blue boxes indicate negative controls. **b** Quantification of angiogenic protein levels of (**a**) 3 Ctrl NSC CM versus 3 SZP NSC CM. Data are shown as level of expression relative to internal control and graphed as mean ± SD; *p < 0.05 according to Mann–Whitney test. **c** Heatmap depicting the levels of angiogenic proteins present in 3 Ctrl NSC CM (#1, #2 and #3), 3 SZP NSC CM (#1, #2 and #3), 3 Ctrl Nsp CM (#1, #2 and #3) and 3 SZP Nsp CM (#1, #2 and #3). Data are shown as average level relative to internal control. **d–g** Quantification of IGBP-2 (**d**), VEGFA (**e**), TIMP-1 (**f**), and NRG1-B1 (**g**), in each of the CM. Data for each cell line (Ctrl #1, #2, #3 and SZP #1, #2, #3) is shown in a correlative order. Values are shown as the level of protein expression relative to internal control and graphed as mean ± SD; *p < 0.05, **p ≤ 0.01 according to Mann–Whitney test. **h–m** Analysis of non-canonic neuroangiogenic factors expression by qPCR in 3 Ctrl NSC and 3 SZP NSC. Data for each cell line (Ctrl #1, #2, #3 and SZP #1, #2, #3) is shown in a correlative order. Data are shown as mean ± SD; *p < 0.05 according to Mann–Whitney test. (**n**) Expression of SEMA3A was evaluated in 3 Ctrl and 3 SZP NSC CM. Data for each cell line (Ctrl #1, #2, #3 and SZP #1, #2, #3) is shown in a correlative order. (**o**) Quantification of protein bands of (**n**) reveal increased SEMA3A expression in SZP NSC. Data are shown as mean ± SD; *p < 0.05 according to Mann–Whitney test

In order to evaluate whether SZP NSC angiogenic secretion is imbalanced, we compared their levels in three SZP NSC CM and three Ctrl NSC CM. Figure 2a shows representative membranes of angiogenesis proteome profiling, depicting the differences in protein abundance as dots for each protein of the array. Quantification of Ctrl and SZP NSC CM protein levels reveal significant differences in the expression of several angiogenic proteins (Fig. 2b). SZP NSC CM have lower concentrations of pro-angiogenic molecules such as Angiogenin, Ang-1, IGFBP-3, PDGF-AA, uPA, and VEGFA, as well as reduced concentrations of anti-angiogenic molecules Endostatin and PEDF. Pro-angiogenic IGFBP-2 and anti-angiogenic PTX3, on the contrary, were up-regulated.

We next wondered if this difference in the CM angiogenic properties was still present when NSC were grown under a differentiation stimuli. We cultured the same SZP NSC and Ctrl NSC as neurospheres (Nsp) in NEM media containing B-27 and N-2 supplements to induce a mixed differentiation^40^. After 4 days of culture CM was collected and the presence of angiogenic proteins was quantified. In contrast to NSC’ CM, Nsp CM presented all the 55 angiogenic proteins assessed, although most of them at very low amounts (Fig. 2c). For the majority of proteins, we did not find a dysregulation in SZP Nsp CM compared to Ctrl Nsp CM, even though they were secreted at similar or even higher levels than equivalent proteins of the NSC CM, such as for IGFBP2 (Fig. 2d). Nevertheless, we still found a significant reduction in the presence of VEGFA in SZP Nsp CM compared to Ctrl Nsp CM (Fig. 2e) and, moreover, the antiangiogenic protein TIMP-1 was increased in SZP Nsp CM compared to Ctrl Nsp CM (Fig. 2f). Noteworthy, NRG1-B1 was dysregulated in SZP Nsp CM compared to Ctrl Nsp CM, a 2.2-fold higher secretion in SZP Nsp CM #3 could be found when comparing to the average Ctrl Nsp CM value (Fig. 2g).

Supporting our results is the fact that post-mortem dorsolateral prefrontal cortex of SZP and adult plasma display reduced expression of VEGFA^17,18^. In line with the possible existence of an autocrine loop for VEGFA signaling, we found that the expression of VEGFA (Fig. 2h) and VEGFA receptors KDR (VEGFR2) and NRP1 is downregulated in SZP NSC compared to Ctrl NSC (Supplementary Figure 2a). Futhermore, abnormalities in the mRNA expression of several isoforms of NRG1 and increment in protein expression have been found in post-mortem dorsolateral prefrontal cortex of SZP^41,42^.

Neuro-vascular development also depends on the so called “non-canonical” signaling pathways^5^. To assess if non-canonical neuro-angiogenic proteins such as Ephrins, NTN1, SLIT2, and SEMA3A were dysregulated, we quantified their mRNA concentration via qPCR in Ctrl and SZP NSC. Pro-angiogenic *EFNA1* (Fig. 2i) was significantly downregulated in SZP NSC while the anti-angiogenic molecule SEMA3A was upregulated (Fig. 2m). *EFNB2, NTN1*, and *SLIT2* have dual roles as they act as either pro- or anti-angiogenic factors, depending on the context imposed by the presence of their specific receptors^43^. Interestingly, we found that expression levels of these three factors were decreased in SZP NSC compared to Ctrl NSC (Fig. 2j–l). SEMA3A has been reported to be increased in SZP^21^; we corroborated this upregulation via Western Blot analysis of Ctrl versus SZP NSC CM (Fig. 2n, o, Supplementary Figure 2b).

### Evaluation of NSC angiogenic capacities in vitro

**Fig. 3:**
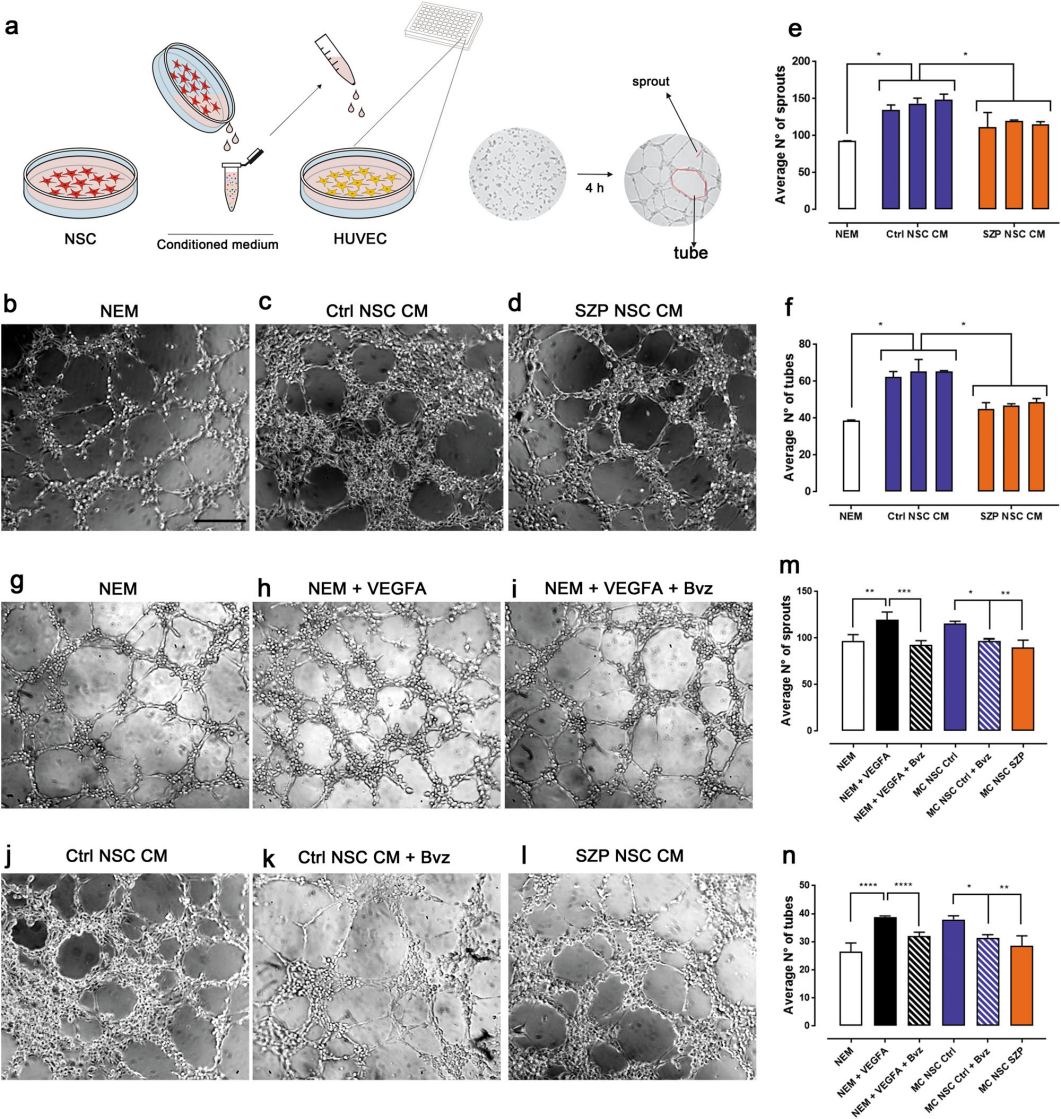
SZP NSC CM induce less angiogenesis and migration in vitro than Ctrl NSC CM. **a** Cartoon representing the experimental design of the tube formation assay. After 48 h, CM was collected from NSC cultures and applied on HUVEC seeded on matrigel coated wells. After 4 h, tubes (closed polygons) and sprouts were counted. **b**–**d** Representative images of tube formation assay when incubating HUVEC on Neural Expansion Media (NEM, used as negative control (**b**), Ctrl NSC CM (**c**) or SZP NSC CM (**d**); Scale bar = 30 µm. **e**–**f** Quantification of average number of sprouts (**e**) or tubes (**f**) formed in each condition. Data for each cell line (Ctrl #1,#2, #3 and SZP #1, #2, #3) is shown in a correlative order control and graphed as mean ± SD with *p < 0.05 according to Kruskal-Wallis test. **g–n** Ctrl NSC CM was incubated with 100 µg/ml of bevacizumab (Bvz) to inhibit VEGFA signaling. **g**–**l** Representative images of tube formation assay when incubating HUVEC on NEM (**g**), NEM plus 50 ng/ml VEGFA (**h**), NEM plus 50 ng/ml VEGFA with 100 µg/ml of bevacizumab (Bvz) inhibitor (**i**), Ctrl NSC CM (**j**), Ctrl NSC CM with 100 µg/ml Bvz (**k**) or SZP NSC CM (**l**). **m**–**n** Quantification of average number of sprouts (**m**) or tubes (**n**) formed in each condition. Data is shown as mean ± SD with *p < 0.05, **p ≤ 0.01 and ***p ≤ 0.001 according to Kruskal–Wallis test

Angiogenesis is controlled by a tight and complex balance between pro-angiogenic and anti-angiogenic signals^44^. Since we found that NSC express several angiogenic proteins and that there is a significant difference in the expression and secretion of many of these factors in Ctrl versus SZP NSC (Fig. 2), we next evaluated Ctrl and SZP NSC CM in a functional assay. Endothelial cells, like HUVEC, are able to rapidly divide and migrate when exposed to pro-angiogenic signals. When cultured on an extracellular matrix, HUVEC form sprouts and tube-like structures (Supplementary Figure 3). We therefore cultured HUVEC in the presence of NSC CM over a four hour period, and evaluated the number of tube and sprouts formed (Fig. 3a). Compared to NEM culture medium (Fig. 3b), Ctrl NSC CM generates more tubes (Fig. 3c). SZP NSC CM on the other hand, was less effective, producing thinner tube structures (Fig. 3d). We demonstrate that the CM of the three Ctrl NSC tested had pro-angiogenic capacities, due to the fact that they induced the formation of more sprouts (Fig. 3e) and tubes (Fig. 3f) when compared to NEM culture medium. The three SZP NSC CM tested on the other hand, failed to significantly induce angiogenesis, relative to the angiogenesis induced by NEM. Overall, SZP NSC CM exhibited a 23% and 37% reduction in number of sprouts and tubes formed, respectively, when compared to that formed by Ctrl NSC CM (Supplementary Figure 3 b, c). In fact, when assaying a dose response by diluting SZP NSC CM into control NSC CM at a range of ratios we corroborated a recovery of angiogenic capacities at a 1:3 dilution (Supplementary Figure 3d–f).

As previously stated, VEGFA has shown to be downregulated in SZP^17,18^. In line with this evidence, we found that SZP NSC express 53% less and secrete 79% less VEGFA than Ctrl NSC (Fig. 2). Thus, we next aimed to investigate the contribution of VEGFA signaling to angiogenesis, displayed by Ctrl NSC CM. We inhibited VEGFA activity by incubating Ctrl NSC CM with bevacizumab (Bvz), a drug that specifically binds to the VEGFA protein, thereby blocking its activity. The incubation of NEM with VEGFA significantly increases both sprout and tube formation; an effect inhibited when adding 100 µg/ml of Bvz (Fig. 3g–n). Likewise, treatment with Bvz of Ctrl NSC CM, diminishes sprout and tube formation in a 10% and 17% respectively, resulting in angiogenic levels similar to the ones induced by SZP NSC CM (Fig. 3j–n).

When evaluating Nsp CM angiogenesis in vitro induction efficiency (Supplementary Figure 3g–l), we observed that none of the six Nsp CM tested generated more sprouts than the control NEM (supplemented) (Supplementary Figure 3m). Despite that, Ctrl Nsp CM presented an increased tube formation when compared to NEM (Supplementary Figure 3n). Even though the average number of sprouts and tubes formed in all conditions was smaller than the one induced by NSC CM, we confirmed that SZP Nsp CM induced less number of sprouts and tubes than that Ctrl Nsp CM (Supplementary Figure 3m, n). In addition to that, the inhibition of VEGFA signaling in Ctrl Nsp CM, produced a decrease in tube, but not in sprout formation (Supplementary Figure 3o–p).

### Evaluation of angiogenesis in vivo

**Fig. 4:**
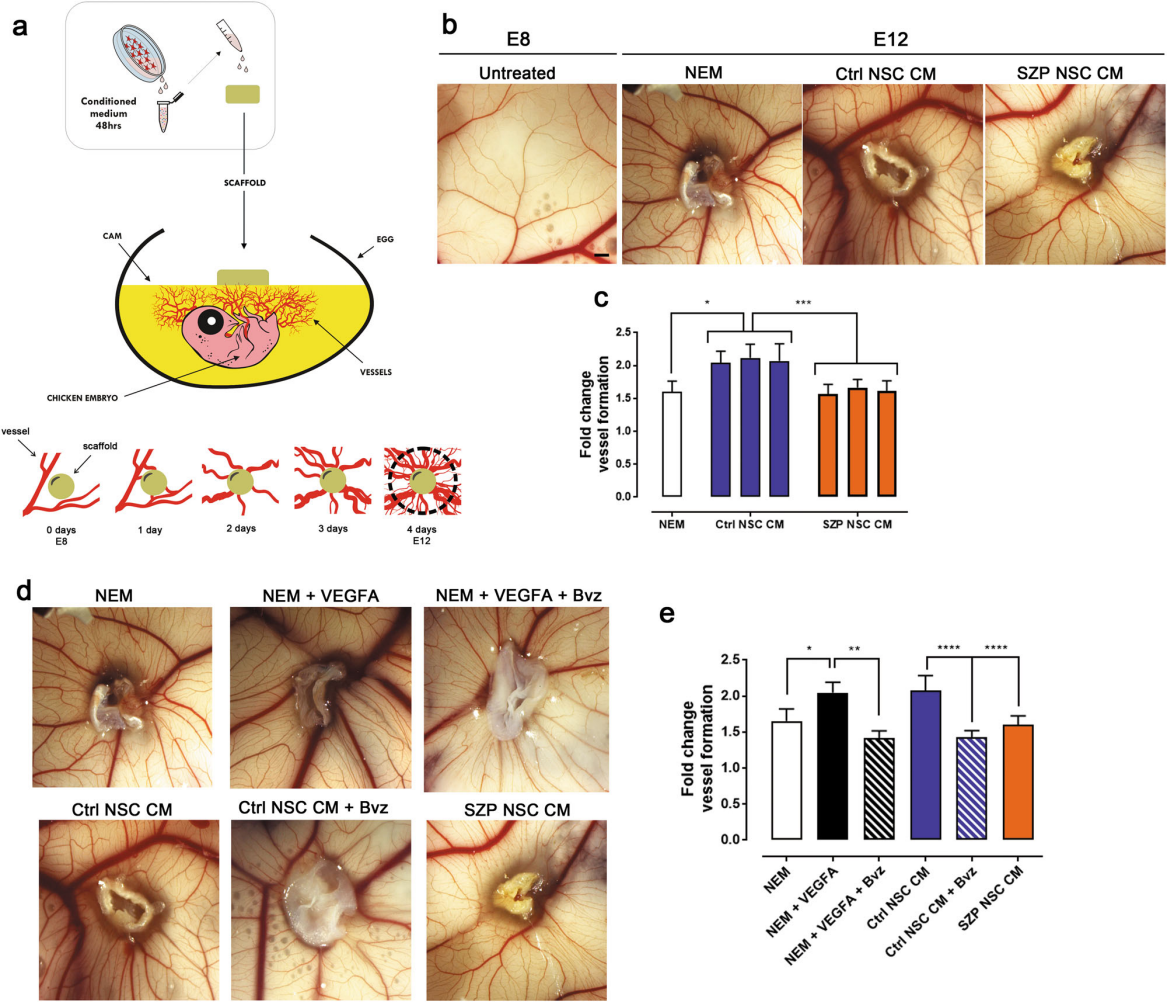
SZP NSC CM induce less angiogenesis in vivo than Ctrl NSC CM. **a** To perform the in vivo angiogenesis assay in the CAM of chicken embryos we added NSC 48 h CM on top of a bio cellulose scaffold. Scaffold was positioned over the CAM at embryonic day 8 (E8). After 4 days, vessels in the perimeter of the scaffold were counted. **b** Representative images of CAM vessels as indicated. Scaffold was filled with NEM as negative control, Ctrl NSC CM and SZP NSC CM. Scale bar = 1 cm. **c** Quantification of number of vessels in each condition at E12, represented as fold change in vessel number compared to E8. Data for each cell line (Ctrl #1, #2, #3 and SZP #1, #2, #3) is shown in a correlative order control and graphed as mean ± SD; * *p* < 0.05 and ***p ≤ 0.001 according to Kruskal–Wallis test. **d**, **e** Ctrl NSC CM was incubated with 100 µg/ml of bevacizumab (Bvz) to inhibit VEGF-A signaling. **d** Representative images of CAM vessels as indicated. Scaffold was filled with NEM, NEM plus 50 ng/ml VEGFA, NEM plus 50 ng/ml VEGFA with 100 µg/ml Bvz, Ctrl NSC CM, Ctrl NSC CM with 100 µg/ml Bvz or SZP NSC CM. **e** Quantification of number of vessels in each condition at E12, represented as fold change in vessel number compared to E8. Data is shown as mean ± SD; *p < 0.05 and ***p ≤ 0.001 according to Kruskal–Wallis test

Due to the complexity of the angiogenesis, we evaluated whether the deficiencies of SZP NSC CM seen in vitro were also observed in vivo. To do such analysis, we used the CAM of the chicken embryo. During embryonic development, CAM vessels are in active angiogenesis. Between E8 and E12, alterations in this process are observable and result in a change in the number of vessels in the CAM. This allows us to assess the effect of pro-angiogenic, anti-angiogenic, or neutral components on angiogenesis (Supplementary Figure 4). We used a biopolymer scaffold filled with a given stimulus and placed it over the CAM at E8. After 4 days we counted the number of vessels that crossed a fixed perimeter around the scaffold (Fig. 4a). Incubating the CAM with NEM culture media resulted in an increase in vasculature; vessel formation was even greater when the scaffold was filled with any of the three Ctrl NSC CM (Fig. 4b). Incubation with SZP NSC CM however resulted in less dense and thinner vasculature compared to the vasculature stimulated by Ctrl NSC CM (Fig. 4b). Quantification of vessel formation revealed that vessel formation with Ctrl NSC CM was on average 25.8% higher than with NEM, and 29.6% higher than with SZP NSC CM; none of SZP NSC CM was unable to increase CAM angiogenesis beyond that induced by NEM (Fig. 4c).

Since VEGFA signaling was crucial for Ctrl NSC CM-induced in vitro angiogenesis we decided to inhibit VEGFA activity by incubating Ctrl NSC CM with Bvz (Fig. 4d–f). The pro-angiogenic effect of Ctrl NSC CM was significantly reduced when the CM was co-treated with 100 µg/ml of the drug, reaching levels similar to the pro-angiogenic effect of SZP NSC CM and NEM (Fig. 4d–f). Interestingly, inhibiting VEGFA in SZP NSC CM had no significant effect (Supplementary figure 4a, b), suggesting that there is an important VEGF reduction perse in SZP NSC CM.

Overall, our results indicate that SPZ NSC express and secrete a lower concentration of trophic factors, and that this leads to alterations in angiogenesis, as confirmed both in vitro and in vivo. Moreover, we demonstrate the important contribution of NSC produced VEGFA in the angiogenic process.

## Discussion

To maintain neuronal aerobic metabolism, the brain requires great vascularization. The BBB is a highly evolved microvasculature system comprised of a vascular lumen lined by brain EC, pericytes in the basal lamina, and associating astrocytic end-feet, microglia, and neurons^10^. This cellular architecture forms functional neurovascular units that regulate molecular trafficking between blood and the brain throughout a lifespan, assuring the delivery of oxygen and nutrients, as well as the removal of carbon dioxide and waste products from neural tissue. This structure is organized and formed during brain development by a combination of signals from the anterior neural tube and recruited EC.

### NSC as a key regulators of angiogenesis

The early neuroepithelium is composed of NSC, from which all subsequent NPC and neuron lineages derive. Hence, the initial neuro-angiogenic niche relies exclusively on a crosstalk between NSC/NPC and EC. Vasculogenesis in the developing brain begins when NSC recruit angioblasts and EC to form the PNVP. VEGFA is thought to be the principle signaling molecule during this process^9^. Here, we show that NSC derived from hiPSC not only produce VEGFA, but also secrete a wide spectrum of other classical pro- and anti-angiogenic factors (Table 1). The latter suggests that NSC can not only recruit EC to promote vasculogenesis and angiogenesis, but that they are also active participators in vessel remodeling and maintenance. Since angiogenesis is a plastic process, NSC may be modulating vessel formation in spatial-temporal correlation with neurogenesis and brain development. In other words, NSC and EC may share forward and feedback signaling present in the “neuro-angiogenic niche”.

We found that most of the angiogenic proteins secreted by NSC have varied impacts on neurogenesis, NSC proliferation and migration, neuroprotection, and cell survival (Table 1). Due to this, and the fact that they form part of the same niche, we propose that all these classical angiogenic proteins have a dual role during brain development and that they should be considered to be neuro-angiogenic factors. Our studies reveal not only an intricate and indispensable relationship between NSC and the developing vasculature, but also the presence of a common molecular mechanism that regulates both blood and nerve vessel wiring.

We also evaluated molecules that were originally described as regulating axon guidance signals, but have lately been thought to be involved in angiogenesis, such as SEMA3A, NTN1, Ephrins, and SLIT2. These proteins are expressed in both EC and NSC in the developing neuro-angiogenic niche and have attractive or repulsive functions, depending on the context and/or microenvironment^43^. As stated before, altering these signals greatly impacts both neurogenic and angiogenic processes, resulting in functional brain deficits. Evaluating NSC angiogenic secretome could lead us to better understand the relation between early alterations in brain vasculature and concomitant neural development, and this relationships role in the development of neurodevelopmental diseases such as schizophrenia. In attempting to tease out the intricacies of this relationship, it is worthwhile to further investigate how multiple growth factors might interact with each other in the neuro-angiogenic niche.

### VEGFA signaling is impaired in SZP

There is growing clinical and experimental data depicting that vascular endothelial dysfunction does occur, at least in a subset of individuals with schizophrenia. To date however, this hypothesis has not been experimentally validated within the context of embryogenesis or linked with an impaired establishment of the neurovascular unit during early brain development in these patients.

When assessing the differences between the angiogenic protein secretions of SZP and Ctrl NSC, we found both pro- and anti-angiogenic factors to be down-regulated (Fig. 2). Importantly, we show that SZP NSC secrete and express less VEGFA than Ctrl NSC. VEGFA is a master regulator of angiogenesis and is the first molecule to be described as a proangiogenic signal derived from the NT. Today, VEGFA is still the most influential factor in early vessel patterning and overall angiogenic processes^8,9,39,45^. Moreover, previous reports show that adult plasma levels of VEGFA and mRNA in post-mortem brains of SZP are reduced with respect to healthy individuals^17,18^. The concordance of these observations with our own study findings is evident. From a therapeutic perspective, our data reveal a strong functional contribution of VEGFA, favoring the use of hiPSC-derived NSC for the study of SZP. On the other hand, our results imply that the VEGFA downregulation reported in adults can actually be traced back to early brain development, as VEGFA influences embryonic vessel formation. Overall, it seems that VEGFA signaling is downregulated in SZP NSC, indicated by a reduction in the concentration of VEGFA receptors KDR and NRP1 (Supplementary Figure 2a). Post-mortem prefrontal cortices of SZP have been shown to exhibit a downregulation of KDR^19^. This reveals the utility of using a stem cell approach to model schizophrenia. By examining conservation of disturbed VEGF signaling in hiPSC- derived NSC, our results can be applied to the establishment of a possible therapeutic strategy.

Other angiogenic proteins that cross-talk with VEGFA were also found to be deregulated. Among them is Angiogenin, a molecule that participates in several aspects of wound healing angiogenesis by increasing rRNA transcription^46^, but has also been reported to be secreted by neurons and mediate motor neuron protection through astroglia^47^. More specifically, Angiogenin seems to be involved in the development of amyotrophic lateral sclerosis and Parkinson’s disease^48,49^. Angiogenin-dependent rRNA transcription is fundamental for VEGFA signaling^50^. Its decrease may therefore act synergistically and could furthermore explain the downregulation of other angiogenic factors^50^. Similarly, PDGF-AA, which promotes VEGFA expression^51^, and uPA, a molecule downstream of VEGFA that initiates angiogenesis by increasing vascular permeability^52^, are also downregulated in SZP NSC CM. Nonetheless, we found that IGFBP-2, which promotes the production and secretion of VEGFA^53^, had increased concentration in SZP NSC CM. While we propose that IGFBP-2 acts as a compensatory mechanism for both angiogenesis and neurogenesis, this apparent disparity needs to be clarified by further studies. Finally, SEMA3A has been reported to be overexpressed in SZP cerebellums and post-mortem prefrontal cortices^21,22^. These observations are related to deficiencies in neuroplasticity and migration of SZP. SEMA3A has also been shown to increase vascular permeability in the brain, suggesting that it could decrease BBB function^54^. We found SEMA3A expression and secretion to be upregulated in SZP NSC. This result is concordant with previous reports on adult SZP, suggesting that there could be an underlying dysfunctional BBB in these patients. Other proteins, such as IGBP3 and Endostatin, which are related to EC survival and/or proliferation^55,56^, were also found to be dysregulated in SZP NSC, most probably contributing to the deterioration of vascular function.

### Dysfunctional induction of migration and angiogenesis of SZP NSC

Due to the deregulation of angiogenic cues produced by the SZP NSC secretome, we examined its induction of angiogenesis. SZP NSC CM failed to induce proper EC migration and tube-like structure formation *in vitro* (Fig. 3). Several angiogenic factors related to EC migration were found to be dysregulated in SZP NSC; non-canonical neuro-angiogenic molecules were also deregulated. A decrease in Angiogenin, uPA, and VEGFA, for example, affected both endothelial and NSC migration (Table 1). Non-canonical angiogenic factors predominately act as chemoattractants or repellents, promoting (or inhibiting) EC migration, and therefore angiogenesis^5^. The pro-angiogenic EFNA1 was downregulated in SZP NSC, whereas the anti-angiogenic SEMA-3A was upregulated in SZP NSC, resulting in a reduction in migration and tube formation by HUVEC (Figs. 2 & 3). Previous works have demonstrated that NTN1 acts as a pro- or anti-angiogenic molecule in a dose dependent manner^35^; NTN1 downregulation could therefore impact angiogenesis positively or negatively, depending on the concentration and presence of receptors, a matter that requires further research.

To assess the complexity of angiogenesis and vessel remodeling beyond EC migration and formation of tube-like structures in vitro, we investigated the induction of angiogenesis in vivo using a CAM assay (Fig. 4). SZP NSC CM significantly impaired new vessel formation. Concordantly, molecules important to vessel remodeling and angiogenesis, such as Ang-1, IGBP-3, uPA, VEGFA, Endostatin, and PEDF were dysregulated in SZP NSC CM (Figs. 2 and 4). As discussed above, VEGFA signaling is importantly impaired in SZP, for which we evaluated the contribution of VEGFA in Ctrl NSC angiogenesis induction using the specific inhibitor bevacizumab. Despite the evident imbalance of other angiogenic factors, VEGFA was still the main contributor to NSC-induced angiogenesis and its downregulation in SZP NSC therefore resulted in great impairment of overall angiogenesis.

### Biomedical significance

Overall, our results favor the prevalent neurodevelopmental hypothesis which stipulates that the presence of developmental abnormalities long before the onset of clinical symptoms results in the emergence of schizophrenia. Studying the mechanisms that modulate angiogenesis is necessary for the development of therapeutic tools for this illness. Here we identify several neuro-angiogenic proteins of NSC origin whose perturbed expression impairs angiogenesis. Our work is a first attempt at examining the neuro-angiogenic mechanisms involved. We are aware that in order to dissect the complex causal relations, more studies are needed in order to mimic the physiology and evaluate the crosstalk with brain microvascular endothelial cells and other main participants of the niche. The utilization of hiPSC in our experiments set the basis for optimization of strategies that aim to manipulate the developing neuro-angiogenic niche.

### hiPSC-derived NSC as a model of early brain development in SZP

Obtaining living cell samples of the human central nervous system, especially that of during development, is limited. Nonetheless, the use of hiPS cells in complex disease studies such as schizophrenia, paved the way to a better understanding of cellular and molecular mechanisms relative to neuronal differentiation and maturation^57^. Although the potential of hiPSC to generate disease models, more specifically schizophrenia, is a powerful technology, the process of neural cell (NSCs, NPCs, and neurons) differentiation from stem cells is complex and must obey a strict sequence of steps^58^. Here we evaluated NSC derived from hiPSC from three different SZP and three different healthy subjects (Fig. 1). SZP NSC did not show any morphological difference with Ctrl NSC, but they presented a defective migration (Supplementary Figure 1), characteristic of schizophrenia phenotype, as has been published before^30^. Moreover, SZP NSC present a clear differential secretome and exhibit a higher secretion of NRG1-B1. The latter has been proposed as a schizophrenia gene^41,42^, thus validating the use of hiPSC derived NSC as model for this disease.

Our results collectively show, for the first time, a probable mechanistic link between a functionally impaired neuro-angiogenic niche and the onset of neurovascular endothelial dysfunction in SZP. Further research is needed to fully explore the relevance of these results within the context of the progression of schizophrenia.

## Electronic supplementary material

Supplementary Information

Supplementary Video 1. Axonal migration of SZP Nsp

Supplementary Video 2. Axonal migration of SZP Nsp
