# Supplementary material for: hiPSC-derived neural stem cells from patients with schizophrenia induce an impaired angiogenesis
Source: Transl Psychiatry. 2018 Feb 22;8:48. doi: 10.1038/s41398-018-0095-9 (PMC5821759; doi:10.1038/s41398-018-0095-9)
Supplement: Supplementary file 1 — Supplementary Information [file 41398_2018_95_MOESM1_ESM.docx]

**Supplementary Information**

Includes:

Supplementary Table 1, Supplementary Table 2 and Supplementary Table 3, with information of iPSC lines, antibodies and PCR primers used in this work, respectively.

Supplementary Figure 1

Supplementary Figure 2

Supplementary Figure 3

Supplementary Figure 4

Supplementary Video Legend 1 and 2

Supplementary Video 1. mp4

Supplementary Video 2. mp4

Supplementary Table 1. hiPSC lines used in this study.

Supplementary Table 2. Antibodies used for Immunofluorescence and Western Blot

| Antibody | Catalog #, Supplier |
| --- | --- |
| SEMA3A | sc-74554, Santa Cruz Biotechnology, Santa Cruz, CA, USA |
| SLIT2 | sc-514499, Santa Cruz Biotechnology, Santa Cruz, CA, USA |
| Nestin | RA22125, Neuromics, Edina, MN, USA |
| Pax6 | sc-81649, Santa Cruz Biotechnology, Santa Cruz, CA, USA |
| Alexa Fluor 546 | A-11003, Invitrogen, Carlsbad, CA, USA |
| Alexa Fluor 488 | A-11008, Invitrogen, Carlsbad, CA, USA |

Supplementary Table 3. Primers used for qPCR amplification

| Gene | Forward 5’-3’ | Reverse 5’-3’ |
| --- | --- | --- |
| *VEGFA* | CTCTACCTCCACCATGCCAAG | AGACATCCATGAACTTCACCACTTC |
| *EFNA1* | AGCCTCAAAACGGGTCAGTA | CATGCCTGCACAGCTTGTTT |
| *EFNB2* | AGGTGCCCTTTAGCCAGAT | ACCAAAGTGCTGTGCTACCT |
| *NTN1* | TGCAAGAAGGACTATGCCGTC | GCTCGTGCCCTGCTTATACAC |
| *SLIT2* | TGTGAGCCATGCCACAAGAA | AACGCATTGATGGGCAAGCA |
| *SEMA3A* | TGGCCAACAGCTCCAGTTAT | TTCCAGCCCACTTGCATTCA |
| *KDR* | TCATGCACGGCATCTGGGAAT | GCACAGCCAAGAACACTGCAT |
| *NRP1* | AGCCTGCAACTTGGGAAACT | TGGTTACCAGGCGGATGTTT |
| *GAPDH* | CAAGAAGGTGGTGAAGCAGGC | CCACCACCCTGTTGCTGTAG |
| *B2M* | GCTATCCAGCGTACTCCAAA | GAAAGACCAGTCCTTGCTGA |
| *18S* | GGGCCCGAAGCGTTTACTTT | TTGCGCCGGTCCAAGAATTT |

**Supplementary Figures**


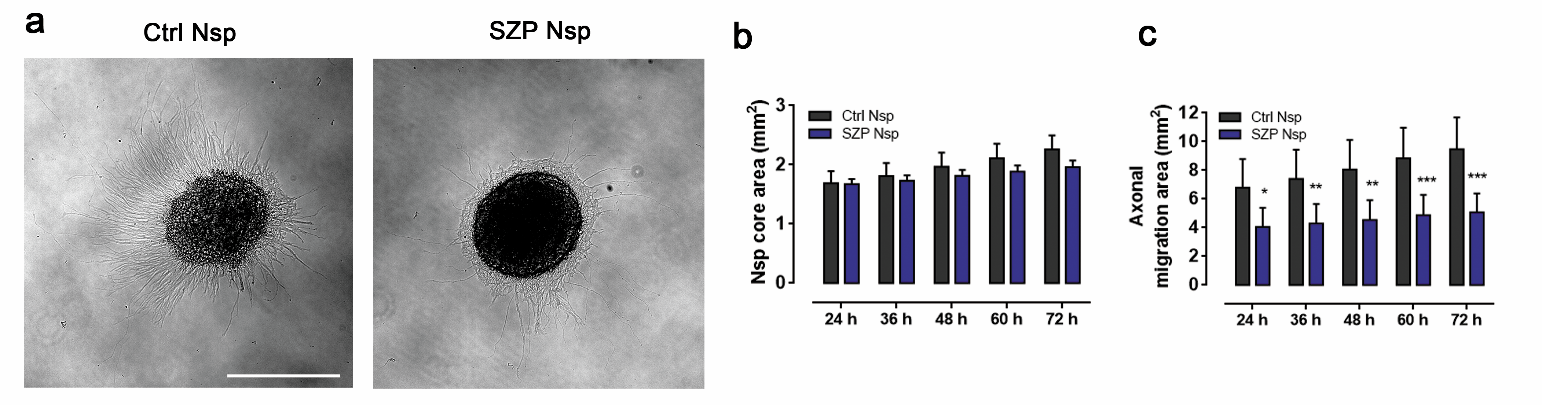


**Supplementary Figure 1. SZP NSC present a defective migration**. (**a**) Representative images of neurosphere (Nsp) migration assay performed to 3 Ctrl NSC and 3 SZP NSC derived Nsp; scale bar = 400 µm (**b**) Measurement of Nsp core area showed no significant difference between Ctrl and SZP Nsp over time. (**c**) The axonal migrating area was significantly smaller in SZP Nsp compared to Ctrl Nsp, over time. Data is shown as mean ± SD; * p < 0.05, ** p ≤ 0.01, *** p ≤ 0.001 according to ANOVA.


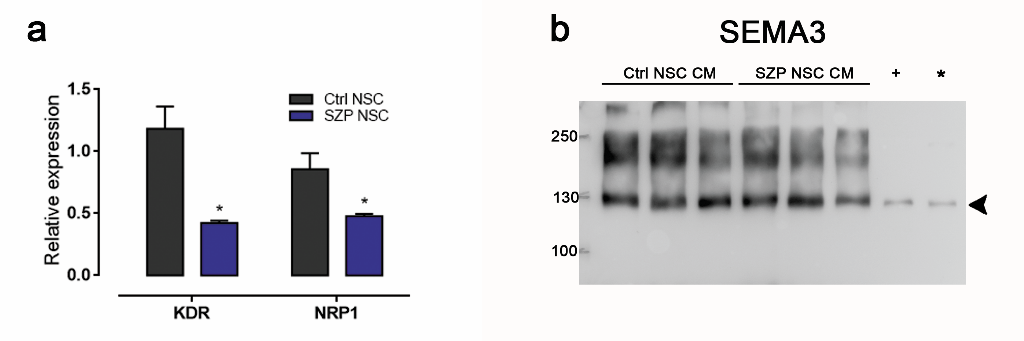


**Supplementary Figure 2. Angiogenic factor expression of SZP NSC**. (**a**) Quantification of of VEGFA receptors KDR and Neuropilin1 expression on 3 Ctrl NSC and 3 SZP NSC by qPCR. Data is shown as mean ± SD; * p < 0.05 according to Mann-Whitney test. (**b**) Expanded information of Figure 2. SEMA3 western blot membrane of 3 Ctrl and 3 SZP NSC; DAOY (+) and Ctrl NSC (*) cell protein extracts were used as positive controls.

**
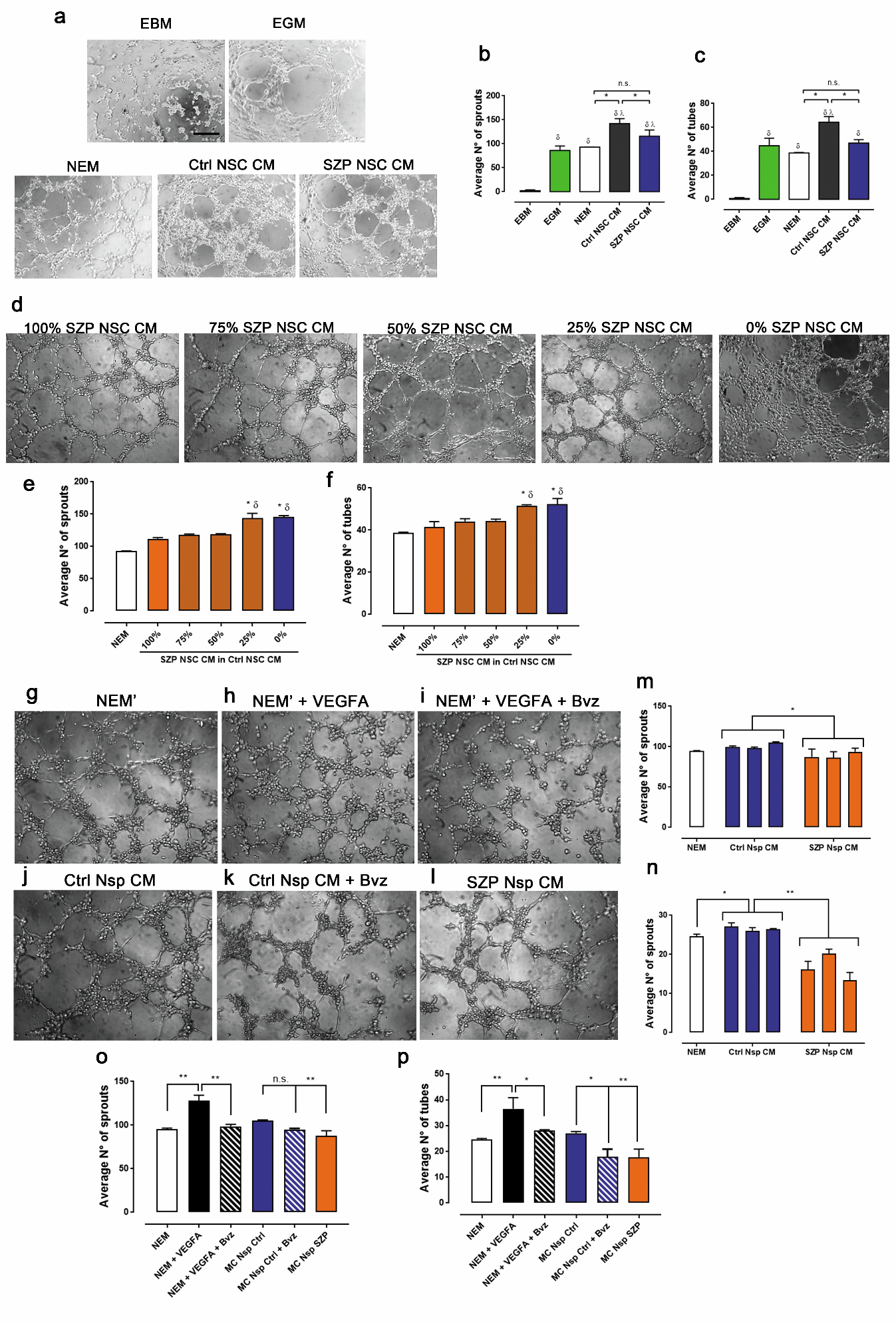
Supplementary Figure 3.SZP NSC CM induced an impaired angiogenesis *in vitro*.** (**a**) For tube formation assay, HUVEC were seeded in Endothelial Basal Medium (EBM) and Endothelial Growth Medium (EGM) to verify normal basal tube and sprout formation. Scale bar = 30 µm. (**b** - **c**) Incubation on EGM, NEM, Ctrl NSC CM and SZP NSC CM produced more sprout and tube formation than HUVEC incubated on EBM. Ctrl NSC CM induced more tube and sprouts than EGM and was more angiogenic than SZP NSC CM. Data is shown as mean of N=3 ± S.D. *, δ (respect to EBM) and λ (respect to EGM) = p < 0.05 according to Kruskal-Wallis test. (**d-f**) SZP NSC CM was diluted in Ctrl NSC CM, were 100% means only SZP NSC CM and 0% means only Ctrl NSC CM. (**d**) Representative images of tube formation assay when incubating HUVEC with different SZP dilutions. (**e**) Quantification of average number of sprouts (**e**) or tubes (**f**) formed in each condition. Data is shown as mean ± SD with * (respect to NEM) and δ (respect to 100%) p < 0.05 according to Kruskal-Wallis test. (**g-l**) Representative images of tube formation assay when incubating HUVEC on NEM’ (NEM with differentiation supplements. (**g**), NEM’ plus 50 ng/ml VEGFA (**h**), NEM’ plus 50 ng/ml VEGFA with 100 µg/ml of Bvz (**i**), Ctrl Nsp CM (**j**), Ctrl Nsp CM with 100 µg/ml Bvz (**k**) or SZP Nsp CM (**l**). (**m**-**n**) Quantification of average number of sprouts (**m**) or tubes (**n**) formed in each condition. Data for each cell line (Ctrl #1,#2, #3 and SZP #1,#2,#3) are shown in a correlative order and graphed as mean ± SD with * p < 0.05 according to Kruskal-Wallis test. (**o-p**) Quantification of average number of sprouts (**o**) or tubes (**p**) formed in each condition. Data is shown as mean of N=3 ± SD with * p < 0.05 according to Kruskal-Wallis test.


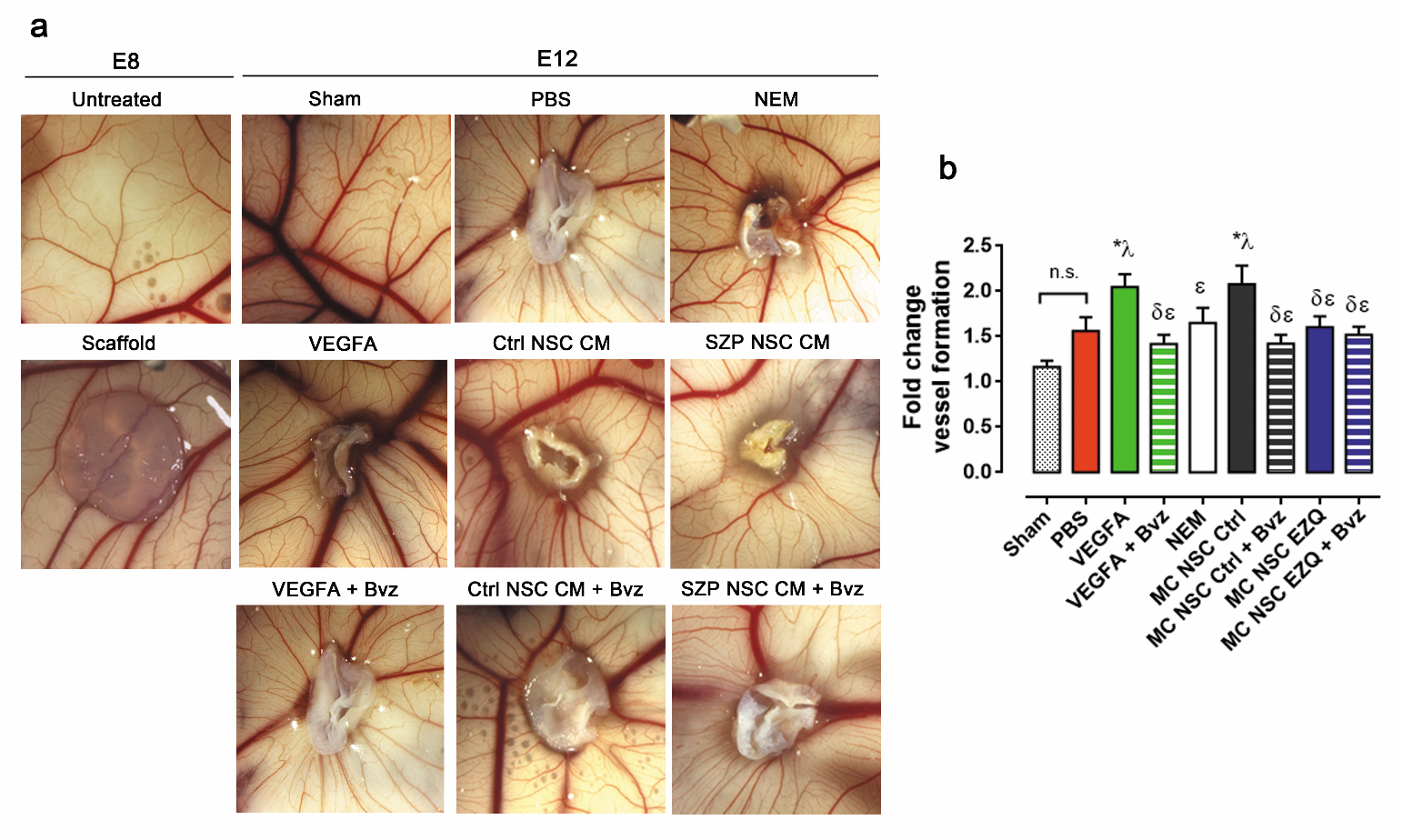


**Supplementary Figure 4. Expanded data of Figure 4.** (**a**) For CAM assay, embryos were photographed at E8 to assess initial vessel number. A scaffold filled with the different stimuli was placed on top of the CAM. (**b**) Quantification of vessels on E12 as fold change respect to E8. No significant difference between Sham (without scaffold) and PBS was found. Fold change of vessel formation is significantly higher for positive control VEGF and Ctrl NSC CM when compared to PBS. * (respect to Sham), λ (respect to PBS), ε (respect to VEGF) and δ (respect to Ctrl NSC CM) = p < 0.05 according to Kruskal-Wallis test.

Supplementary Video Legend

Supplementary Video 1. Video shows axon migration of representative Ctrl Nsp from 24 to 72 h after plating.

Supplementary Video 2. Video shows axon migration of representative SZP Nsp from 24 to 72 h after plating.
